# Prevalence of disorders recorded in Cavalier King Charles Spaniels attending primary-care veterinary practices in England

**DOI:** 10.1186/s40575-015-0016-7

**Published:** 2015-04-18

**Authors:** Jennifer F Summers, Dan G O’Neill, David B Church, Peter C Thomson, Paul D McGreevy, David C Brodbelt

**Affiliations:** Royal Veterinary College, Hawkshead Lane, Hatfield, Hertfordshire, AL9 7TA UK; Faculty of Veterinary Science, University of Sydney, New South Wales, 2006 Australia

**Keywords:** Prevalence, Canine, Cavalier King Charles spaniel, CKCS, Electronic patient record, EPR, Primary-care practice, VetCompass

## Abstract

**Background:**

Concerns have been raised over breed-related health issues in purebred dogs, but reliable prevalence estimates for disorders within specific breeds are sparse. Electronically stored patient health records from primary-care practice are emerging as a useful source of epidemiological data in companion animals. This study used large volumes of health data from UK primary-care practices participating in the VetCompass animal health surveillance project to evaluate in detail the disorders diagnosed in a random selection of over 50% of dogs recorded as Cavalier King Charles Spaniels (CKCSs). Confirmation of breed using available microchip and Kennel Club (KC) registration data was attempted.

**Results:**

In total, 3624 dogs were recorded as CKCSs within the VetCompass database of which 143 (3.9%) were confirmed as KC-registered via microchip identification linkage of VetCompass to the KC database. 1875 dogs (75 KC registered and 1800 of unknown KC status, 52% of both groups) were randomly sampled for detailed clinical review. Clinical data associated with veterinary care were recorded in 1749 (93.3%) of these dogs. The most common specific disorders recorded during the study period were heart murmur (541 dogs, representing 30.9% of study group), diarrhoea of unspecified cause (193 dogs, 11.0%), dental disease (166 dogs, 9.5%), otitis externa (161, 9.2%), conjunctivitis (131, 7.4%) and anal sac infection (129, 7.4%). The five most common disorder categories were cardiac (affecting 31.7% of dogs), dermatological (22.2%), ocular (20.6%), gastrointestinal (19.3%) and dental/periodontal disorders (15.2%).

**Discussion and conclusions:**

Study findings suggest that many of the disorders commonly affecting CKCSs are largely similar to those affecting the general dog population presented for primary veterinary care in the UK. However, cardiac disease (and MVD in particular) continues to be of particular concern in this breed.

**Further work:**

This work highlights the value of veterinary practice based breed-specific epidemiological studies to provide targeted and evidence-based health policies. Further studies using electronic patient records in other breeds could highlight their potential disease predispositions.

## Lay summary

Concerns have been raised regarding breed-related health issues in purebred dogs, but reliable information on the extent of particular problems within individual breeds remains sparse. This study describes the health disorders most frequently recorded across a large group of Cavalier King Charles spaniels (CKCSs) presented for primary health care in England.

Study dogs were identified from an extensive archive of electronic patient records held by the VetCompass animal surveillance programme. Disorder frequencies were obtained by reviewing all clinical health information for these dogs, as recorded by primary-care veterinary practitioners between July 2007 and July 2013.

In the 1875 CKCSs randomly selected for detailed clinical review the most common disorders recorded were heart murmur, diarrhoea, dental disease, otitis externa, conjunctivitis and anal sac infection. The most common disorder categories were cardiac, dermatological, ocular, gastrointestinal and periodontal disorders.

Disorders commonly reported in study CKCSs appeared similar to those affecting the general, vet-visiting dog population the UK. However, cardiac diseases and Mitral Valve disease (MVD) in particular, continue to be of particular concern in this breed.

This work demonstrates the value of veterinary practice based, breed-specific studies in highlighting common problems and potential disease predispositions in popular dog breeds. This knowledge is invaluable to vets, dog owners, breeders and can be used to prioritise particular purebred dog health issues for investigation and targeted action.

## Background

Health problems in purebred dogs have long been of concern for those with an interest in canine health and welfare [1], and have remained topical in recent years [2,3]. Certain canine breeds are reportedly predisposed to particular problems associated with genetic transmission of deleterious traits, including a range of conformational and non-conformational disorders [4,5]. Clarification of the scale of the problem within existing canine breed populations has been hampered by a lack of data on the prevalence of so-called breed-associated disorders among existing canine populations [6-8].

Reliable information on the conditions commonly affecting dogs of certain breeds is extremely important to many stakeholders concerned with canine health and welfare. This information is invaluable to veterinary practitioners making clinical decisions about, or advising clients on matters related to, purebred dogs, while awareness of particular breed-associated problems can help potential or existing owners and breeders to make informed choices when purchasing, caring for or breeding from these dogs [9]. Breed-specific, baseline estimates of disease prevalences are also vital for animal health and welfare researchers, to help effectively channel available resources for canine health research within breeds of particular interest to individual funding bodies (and to monitor the effects of any changes implemented) [10].

Identification of the information gaps regarding canine disease prevalence in the UK [11] has helped to stimulate progress in this domain; however reliable prevalence estimates for disorders within many breeds in the UK remain sparse [5]. Many existing estimates are difficult to apply to UK situations due to geographical differences [12,13] or are subject to one or more potential biases associated with small study groups, use of study samples obtained solely from referral-hospital clinical populations [7,14] and the limitations involved when conducting questionnaire-based surveys [15] or retrospective analysis of insurance data [16-18]. Recently published work by O’Neill et al. [11] used large volumes of electronic patient record (EPR) healthcare data from UK primary-care veterinary clinics participating in the VetCompass animal health surveillance project [19] to estimate the prevalence of disorders diagnosed in the general canine population presented. Using a ‘big-data’ approach, this study was able to report the most common disorders diagnosed among study dogs, and enable comparison between purebreds and crossbreds as well as between certain popular breed groups.

Despite declining Kennel Club (KC) registrations for the breed in recent years, the Cavalier King Charles Spaniel (CKCS) continues to rank consistently in the top 20 UK Kennel Club (KC) registered breeds, with 5,145 new registrations in 2013 [20]. Disorders with an inherited (thus breed-related) basis in CKCSs include mitral valve insufficiency (MVI/MVD) [21,22] and congenital keratoconjunctivitis sicca and ichthyosiform dermatosis syndrome (CKCSID), commonly known as dry-eye-curly-coat-syndrome [23-25]. More recently, concern has been raised regarding the possible emergence of syringomyelia as a prominent disease within the breed [26,27]. However, the nature and prevalence of the most common disorders affecting CKCSs have not been reliably determined from a large study group presented for primary veterinary care. Such information is critical to assist diagnostic decision-making in individual dogs and to highlight priority areas for the improvement of health and welfare in the breed as a whole [7,28].

Accepting the KC-registration database as the gold standard, it is possible to confirm pedigree and breed status of dogs identified with sub-dermal microchips by cross-matching them within the KC-registration and VetCompass databases [29]. Such confirmation of pedigree could improve the validity of breed-specific studies and allow comparison between registered and non-registered individuals within a breed.

This study aimed to evaluate health in CKCS dogs attending primary-care veterinary clinics in England. Objectives of the study were to:

- Describe breed-specific demography (e.g. bodyweight, gender, colour and neutering status) and the most frequently recorded disorders in a large sample of CKCSs presented to primary-care practice.
- Evaluate linkage between the KC-registration database and the VetCompass database using sub-dermal identification microchip data.
- Compare the prevalence of the most common disorders of KC-registered CKCSs with CKCSs of unknown KC-registration status, to test the hypothesis that there is no difference between these subgroups.

## Results and discussion

A search of all dogs registered at clinics contributing data to the VetCompass database between 1st September 2009 and 9th July 2013 identified a total of 3624 dogs with breed recorded as CKCS. These dogs presented to 151 individual clinics, distributed between the north-east and south of England; 92 (61%) and 59 (39%) clinics were members of the Medivet and Vets4Pets groups, respectively.

Microchip data were available in 1692 (46.7%) of the 3624 identified CKCSs. It was possible to crosslink microchip data with KC-registration details in 143 of these dogs; this represented 8.5% of all identified CKCSs with microchip data, and 3.9% of all identified CKCSs. The remaining 3481 dogs were classified as of unknown KC-registration status.

The 52% randomly selected sample of all identified CKCSs totalled 1875 dogs: 1800 with unknown and 75 with confirmed KC-registration status. These 1875 dogs were seen at 109 individual clinics during the study period, including 90 (83%) Medivet and 19 (17%) Vets4Pets sites located from north-east to southern England. All study dogs had data available in at least one demographic field of interest. A total of 1749 (93.3%) of the study dogs had at least one information entry associated with a clinical encounter recorded in their EPR from the study period. It was not possible to determine if the 126 dogs without a clinical healthcare entry in their records had died, left the care of the practice, received care at a non-VetCompass practice or were genuinely not presented for any healthcare visits or concerns during the study period. These dogs were retained in the study for description of study dog demography (n = 1875 in these calculations) but were not included within the denominator for disorder prevalence estimates or disorder recording frequencies (i.e. n = 1749 in these calculations).

Of the study dogs (n = 1875), 50.9% were male (Table 1). Based on the maximum bodyweight value available in dogs aged 9 months or older (n = 1307), median bodyweight was 10.5 kg (range 2.8-27.3 kg). Median ages at first and last consultation were 4.0 and 5.25 years, respectively (ranges one month - 17.2 years for both age measures). The most frequent coat colours were Blenheim (44.3%) and tri-colour (30.8%) (Table 1). Of the 1521 dogs with more than one clinical data entry, median time contributed to the study was 1.3 years (range 1 day to 3.6 years).

**Table 1:** **Sex and coat colour of dogs in the study sample**

**Variable**	**Variable Category**	**Number of dogs**	**% of study group**
**Sex**	Male	965	51.47
	Female	905	48.27
	Unspecified	5	0.27
**Coat colour**	Blenheim	828	44.16
	Tri-Colour	576	30.72
	Ruby	227	12.11
	Black and White	103	5.49
	Other solid colour	64	3.41
	Black and Tan	39	2.08
	Other mixed colour	37	1.97
	Unspecified	1	0.05
***Totals per variable***		***1875***	***100.0%***

*Distribution of sex and coat colour in the 1875 Cavalier King Charles Spaniels randomly selected for detailed review of clinical notes (percentages given to 2 decimal places).*

Overall, the specific disorders affecting the greatest proportions of dogs during the study period were heart murmur (541 study dogs; 30.9% of sample group), diarrhoea of unspecified cause (193; 11.0%), dental disease (166; 9.5%), otitis externa (161; 9.2%) and conjunctivitis (131; 7.5%) (Table 2).

**Table 2:** **The 30 specific disorders most frequently recorded in study dogs**

**Specific diagnosis recorded**	**Frequency rank**	**Total dogs diagnosed** ***(KC-registered; KC registration unknown)***	**Prev** ^**a**^ **(%)**	**95% CI for prev** ^**b**^ **(%)**
Heart murmur	*1*	541 *(9; 532)*	30.9	(28.8 - 33.1)
Diarrhoea (unspecified cause)	*2*	193 *(11; 182)*	11.0	(9.7 –12.6)
Dental disease	*3*	166 *(4; 162*)	9.5	(8.2 –11.0)
Otitis externa	*4*	161 *(8; 153)*	9.2	(7.9 –10.7)
Conjunctivitis	*5*	131 *(7; 124)*	7.5	(6.3 –8.8)
Anal sac infection	*6*	129 *(5; 124)*	7.4	(6.2 –8.7)
Heart (cardiac) disease (unspecified)	*7*	128 *(0; 128)*	7.3	(6.2 –8.6)
Corneal disorder (unspecified)	*8*	114 *(9; 105)*	6.5	(5.5 –7.8)
Periodontal disease	*9*	98 *(0; 98)*	5.6	(4.6 –6.8)
Mitral valve disorder	*10*	88 *(0; 88)*	5.0	(4.1 –6.2)
Umbilical hernia	*11*	72 *(8; 64)*	4.1	(3.3 –5.2)
Flea infestation	*12*	64 *(4; 60)*	3.7	(2.9 –4.7)
Anal sac impaction	*13*	63 *(4; 59)*	3.6	(2.8 –4.6)
Cutaneous mass lesion (unspecified)	*14*	62 *(1; 61)*	3.5	(2.8 –4.5)
Keratoconjunctivitis sicca (Dry Eye, KCS)	*15*	61 *(2; 59)*	3.5	(2.7 –4.5)
Gastroenteritis	*16*	59 *(1; 58)*	3.4	(2.6 –4.3)
Patellar luxation	*17*	58 *(2; 56)*	3.3	(2.6 –4.3)
Otitis (unspecified)	*18*	48 *(3; 45)*	2.7	(2.1 –3.6)
Osteoarthritis (osteoarthrosis, DJD^c^)	*19*	46 *(1; 45)*	2.6	(2.0 –3.5)
Colitis	*20*	44 *(4; 40)*	2.5	(1.9 –3.4)
Cataract	*21*	43 *(0; 43)*	2.5	(1.8 –3.3)
Otodectes cynotis infestation	*22*	42 *(3; 39)*	2.4	(1.8 –3.2)
Urinary tract infection	*23*	42 *(4;38)*	2.4	(1.8 –3.2)
Gastritis	*24*	41 *(3; 38)*	2.3	(1.7 –3.2)
Arthropathy(joint disorder)(unspecified)	*25*	39 *(2; 37)*	2.2	(1.6 –3.0)
Pyoderma	*26*	37 *(1; 36)*	2.1	(1.5 –2.9)
Enteritis	*27*	33 *(2; 31)*	1.9	(1.3 –2.7)
Pancreatitis	*28*	32 *(1; 31)*	1.8	(1.3–2.6)
Syringomyelia (SM)	*29*	29 *(1; 28)*	1.7	(1.2–2.4)
ICT^d^ (Kennel Cough)	*30*	29 *(1; 28)*	1.7	(1.2–2.4)

*The 30 most frequently recorded specific disorders in study dogs with available clinical notes (n = 1749) showing frequency ranks and numbers of dogs affected. Calculated prevalence estimates (%) for these conditions in the UK CKCS population are presented with 95% confidence intervals.*

^a^Prev, Prevalence estimate; ^b^95% CI for prev, 95% confidence interval for prevalence estimate; ^c^DJD, Degenerative joint disease; ^d^ICT, Infectious canine tracheobronchitis.

**Figure 1:**
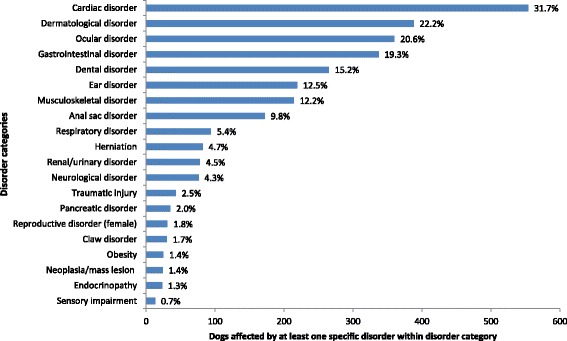
The 20 most frequent disorder categories affecting a randomly selected subset of Cavalier King Charles Spaniels identified in VetCompass data (%’s given represent proportions of the 1749 dogs in the sample (with available clinical notes) affected by at least one specific disorder in this category during the study period).

Cardiac disorders affected the greatest number of individual study dogs; 31.7% of the 1749 randomly selected study dogs with clinical EPR entries had at least one recorded disorder within this category during the study period. The next most frequent categories were dermatological disorders (22.2% of 1749 dogs), ocular disorders (20.6%), gastrointestinal disorders (19.3%) and dental/periodontal disease (15.2%) (Figure 1).

Table 3 presents recording frequencies for disorder groups and specific disorders within the top 10 disorder categories affecting study dogs, overall and by KC-registration status subgroup. Heart murmurs accounted for most of the specific disorders recorded within the cardiac disease category; 541 dogs had a recorded murmur of specified or unspecified grade and murmurs accounted for 71% of cardiac disorders during the study period. Ectoparasite infestation (largely by fleas and mites) accounted for the greatest number of disorder recordings in the dermatological category (27% of category disorders recorded). The largest group of ocular disorders were corneal diseases (43%), with unspecified corneal problems and KCS most frequently recorded. Generalised gastroenteropathies (most frequently diarrhoea of unspecified cause, followed by gastroenteritis) accounted for 89% of recorded disorders within the gastrointestinal disorder category.

**Table 3:** **Breakdown of specific disorder and disorder group recording frequencies within the top 10 disorder categories affecting study CKCSs**

**Disorder category (No. dogs; % of total dogs affected)**	**Disorder groups within category**	**Total disorder recordings in group (% of all in disorder category)**	**Specific disorder**	**Total dogs with specific disorder recorded** ***(KC-registered; KC registration status unknown)***
**Cardiac disorders** (554; 31.7%)	Heart (cardiac) murmur	541 (69.2)	*Heart (cardiac) murmur*	*541 (9; 532)*
	Heart (cardiac) disease	128 (16.4)	*Heart (cardiac) disease (unspecified)*	*128 (0; 128)*
	Cardiac valve disorder	92 (11.8)	*Heart (cardiac) valve disorder*	*4 (0; 4)*
			*Mitral valve disorder*	*88 (0; 88)*
	Cardiopulmonary arrest	8 (1.0)	*Cardiopulmonary arrest*	*8 (0; 8)*
	Cardiomyopathy	4 (0.5)	*Cardiomyopathy finding*	*4 (0; 4)*
	Cardiac rhythm disturbance	3 (0.4)	*Atrial fibrillation*	*1 (0; 1)*
			*Cardiac conduction disorder - Ventricular Premature Complexes (VPCs)*	*1 (0; 1)*
			*Ventricular tachycardia (VT)*	*1 (0; 1)*
	Congenital cardiac malformation	3 (0.4)	*Heart (cardiac) anomaly, congenital - Atrial septal defect (ASD)*	*1 (0; 1)*
			*Patent ductus arteriosus (PDA)*	*2 (0; 2)*
	Pericardial disease	2 (0.3)	*Pericardial disorder finding*	*1 (0; 1)*
			*Pericardial effusion*	*1 (0; 1)*
	Cardiac injury/damage	1 (0.1)	*Chordae tendinae rupture*	*1 (0; 1)*
**Dermatological disorders** (388; 22.2%)	Ectoparasite infestation	142 (26.7)	*Parasite infestation - fleas*	*64 (4; 60)*
			*Parasite infestation - Acariasis (mites)*	*50 (4; 46)*
			*Parasite infestation - ticks*	*27 (5; 22)*
			*Parasite infestation - Pediculosis (lice)*	*1 (0; 1)*
			*Parasite infestation- unspecified*	*1 (0; 1)*
	Cutaneous mass lesion/neoplasia	96 (18.1)	*Mass lesion - skin/cutaneous (unspecified)*	*62 (1; 61)*
			*Papilloma*	*14 (1; 13)*
			*Mass-lesion - Injection reaction*	*5 (0; 3)*
			*Mass lesion - facial, lip*	*4 (0; 4)*
			*Callus*	*3 (0; 3)*
			*Acrochordon (Fibroepithelial polyp, Skin tag)*	*2 (0; 2)*
			*Granuloma*	*2 (0; 2)*
			*Histiocytoma (cutaneous, skin)*	*2 (0; 2)*
			*Mass lesion - facial*	*1 (0; 1)*
			*Melanoma*	*1 (0; 1)*
	Cutaneous infection	76 (14.3)	*Pyoderma*	*37 (1; 36)*
			*Intertrigo*	*20 (1; 19)*
			*Abscess*	*15 (2; 13)*
			*Acne - Canine acne*	*2 (0; 2)*
			*Anal furunculosis*	*1 (0; 1)*
			*Folliculitis and furunculosis - of muzzle*	*1 (0; 1)*
	Dermatitis	51 (9.6)	*Pododermatitis*	*26 (0; 26)*
			*Pyotraumatic dermatitis, acute moist*	*12 (2; 10)*
			*Acral lick dermatitis (granuloma)*	*6 (1; 5)*
			*Malassezia dermatitis*	*3 (0; 3)*
			*Dermatitis - seborrheic*	*2 (0; 2)*
			*Dermatitis and/or panniculitis, (pyo)granulomatous - bacterial*	*1 (0; 1)*
			*Dermatitis and/or panniculitis, (pyo)granulomatous - fungal*	*1 (0; 1)*
	Hypersensitivity (allergic) skin disorder	48 (9.0)	*Hypersensitivity (allergic) skin disorder - unspecified*	*24 (2; 22)*
			*Hypersensitivity (allergic) skin disorder - atopic dermatitis*	*18 (2; 16)*
			*Hypersensitivity (allergic) skin disorder - flea bite hypersensitivity*	*4 (0; 4)*
			*Hypersensitivity (allergic) skin disorder - contact hypersensitivity*	*1 (0; 1)*
			*Hypersensitivity (allergic) skin disorder - urticaria*	*1 (0; 1)*
	Wound/skin trauma	44 (8.3)	*Laceration*	*15 (2; 13)*
			*Wound*	*18 (0; 18)*
			*Wound, infected*	*4 (0; 4)*
			*Post-operative complication - wound infection*	*3 (0; 3)*
			*Post-operative complication - wound seroma*	*3 (0; 3)*
			*Post-operative complication - self-trauma*	*1 (0; 1)*
	Cutaneous cyst	23 (4.3)	*Skin (cutaneous) cyst*	*20 (1; 19)*
			*Interdigital cyst (dogs)*	*3 (0; 3)*
	Foreign body - cutaneous (skin)	18 (3.4)	*Foreign body - cutaneous (skin)*	*18 (1; 17)*
	Alopecia	12 (2.3)	*Alopecia*	*12 (0; 12)*
	Insect bite/sting	12 (2.3)	*Hymenoptera stings (bee, wasp)*	*8 (0; 8)*
			*Insect bite(s)*	*4 (0; 4)*
	Sebaceous gland disorder	4 (0.8)	*Sebaceous gland disorder (unspecified)*	*3 (0; 3)*
			*Sebaceous adenitis*	*1 (0; 1)*
	Cellulitis	2 (0.4)	*Cellulitis*	*2 (0; 2)*
	Dermatophytosis (Ringworm)	1 (0.2)	*Dermatophytosis (Ringworm)*	*1 (0; 1)*
	Footpad hyperkeratosis	1 (0.2)	*Footpad hyperkeratosis*	*1 (0; 1)*
	Pemphigus foliaceus	1 (0.2)	*Pemphigus foliaceus*	*1 (0; 1)*
**Ocular disorders** (360; 20.6%)	Corneal disorder/damage	189 (42.6)	*Corneal disorder (unspecified)*	*114 (9; 105)*
			*Keratoconjunctivitis sicca (KCS, Dry Eye)*	*61 (2; 59)*
			*Keratitis - chronic superficial (pannus)*	*8 (0; 8)*
			*Nuclear sclerosis*	*3 (0; 3)*
			*Corneal stromal abscess*	*1 (0; 1)*
			*Foreign body - intra-ocular, corneal*	*1 (0; 1)*
			*Trichiasis*	*1 (0; 1)*
	Conjunctivitis	131 (29.5)	*Conjunctivitis*	*131 (7; 124)*
	Cataract	43 (9.7)	*Cataract*	*43 (0; 43)*
	Eyelid disorder/malformation	30 (6.8)	*Mass lesion - eyelid*	*22 (1; 21)*
			*Neoplasm - eyelid*	*5 (0; 5)*
			*Entropion*	*3 (0; 3)*
	Uveitis	15 (3.4)	*Uveitis*	*15 (0; 15)*
	Ectopic cilia	11 (2.5)	*Distichiasis*	*11 (1; 10)*
	Ophthalmic (eye) injury	9 (2.0)	*Ophthalmic (eye) injury*	*9 (1; 8)*
	Third eyelid/nictitating membrane disorder - prolapsed gland (Cherry eye)	5 (1.1)	*Third eyelid/nictitating membrane disorder-prolapsed gland (Cherry eye)*	*5 (1; 4)*
	Tear duct disorder	4 (0.9)	*Tear duct abnormality*	*4 (0; 4)*
	Ocular/ophthalmic disorder(unspecified)	3 (0.7)	*Ocular/ophthalmic disorder (unspecified)*	*3 (0; 3)*
	Strabismus	1 (0.2)	*Strabismus*	*1 (0; 1)*
	Glaucoma	1 (0.2)	*Glaucoma*	*1 (0; 1)*
	Lens luxation	1 (0.2)	*Lens luxation*	*1 (0; 1)*
	Mass lesion - conjunctival	1 (0.2)	*Mass lesion - conjunctival*	*1 (0; 1)*
**Gastrointestinal disorders** (337; 19.3%)	Gastroenteropathy (includes gastritis, colitis, enteritis, gastroenteritis, enterocolitis)	383 (88.9)	*Diarrhoea*	*193 (11; 182)*
			*Gastroenteritis*	*59 (1; 58)*
			*Colitis*	*44 (4; 40)*
			*Gastritis*	*41 (3; 38)*
			*Enteritis*	*33 (2; 31)*
			*Canine haemorrhagic gastroenteritis (HGE)*	*11 (1; 10)*
			*Enteropathy*	*2 (1; 1)*
	Gastrointestinal infectious disease	15 (3.5)	*Giardiasis*	*12 (0; 12)*
			*Campylobacteriosis*	*1 (0; 1)*
			*Coccidiosis*	*1 (0; 1)*
			*Parvovirus infection*	*1 (0; 1)*
	Foreign body (ingested)	12 (2.8)	*Foreign body - gastric (stomach)*	*6 (0; 6)*
			*Dietary indiscretion - foreign body ingestion*	*3 (0; 3)*
			*Foreign body - intestinal, small*	*2 (0; 2)*
			*Foreign body - oesophageal*	*1 (0; 1)*
	Constipation	5 (1.2)	*Constipation*	*5 (0; 5)*
	Inflammatory bowel disease (IBD)	4 (0.9)	*Inflammatory bowel disease (IBD)*	*4 (0; 4)*
	Parasites -gastrointestinal	4 (0.9)	*Nematode (roundworm) infestation*	*3 (0; 3)*
			*Gastrointestinal helminth infestation (worms)*	*1 (0; 1)*
	Incontinence - faecal	3 (0.7)	*Incontinence - faecal*	*3 (0; 3)*
	Mass or neoplasia - Intestinal	2 (0.5)	*Adenocarcinoma - small intestinal*	*1 (0; 1)*
			*Mass lesion - intestinal*	*1 (0; 1)*
	Rectal prolapse	1 (0.2)	*Rectal prolapse*	*1 (1; 0)*
	Protein-losing enteropathy (PLE)	1 (0.2)	*Protein-losing enteropathy (PLE)*	*1 (0; 1)*
	Megaoesophagus	1 (0.2)	*Megaoesophagus*	*1 (0; 1)*
**Dental/periodontal disorders** (265; 15.2%)	Dental or periodontal disease	264 (93.3)	*Dental disease*	*166 (4; 162*)
			*Periodontal disease*	*98 (0; 98)*
	Retained deciduous tooth	15 (5.3)	*Retained deciduous tooth*	*15 (0; 15)*
	Dental abscess	4 (1.4)	*Abscess - dental (tooth)*	*4 (0; 1)*
**Ear disorders** (219; 12.5%)	Otitis (externa or media)	215 (84.3)	*Otitis externa*	*161 (8; 153)*
			*Otitis (unspecified)*	*48 (3; 45)*
			*Otitis media*	*6 (2; 4)*
	Foreign body (aural)	27 (10.6)	*Foreign body - aural (ear)*	*27 (2; 25)*
	Ear (aural) infection	11 (4.3)	*Ear (aural) infection - fungal*	*8 (0; 8)*
			*Ear (aural) infection - bacterial*	*3 (1; 2)*
	Mass lesion or neoplasia - ear	1 (0.4)	*Adenocarcinoma - aural (ear)*	*1 (0; 1)*
	Inner ear disorder	1 (0.4)	*Tympanic bulla abnormal*	*1 (0; 1)*
**Musculoskeletal disorders** (214; 12.2%)	Arthritis	71 (24.2)	*Osteoarthritis (osteoarthrosis, degenerative joint disease (DJD))*	*46 (1; 45)*
			*Arthritis*	*23 (0; 23)*
			*Polyarthropathy - immune-mediated*	*1 (0; 1)*
			*Rheumatoid arthritis (RA)*	*1 (0; 1)*
	Joint luxation/subluxation	64 (21.8)	*Patellar luxation*	*58 (2; 56)*
			*Joint luxation (unspecified)*	*6 (1; 5)*
	Arthropathy (unspecified)	39 (13.3)	*Arthropathy (Joint disorder)(unspecified)*	*39 (2; 37)*
	Musculoskeletal injury	39 (13.3)	*Musculoskeletal injury (unspecified)*	*21 (0; 21)*
			*Fracture*	*10 (1; 9)*
			*Muscle injury*	*4 (1; 3)*
			*Ligament injury*	*3 (0; 3)*
			*Tendon injury*	*1 (0; 1)*
	Intervertebral disc disorder	21 (7.2)	*Intervertebral disc disorder (unspecified)*	*20 (0; 20)*
			*Intervertebral disc extrusion/herniation/prolapse - cervical*	*1 (0; 1)*
	Spondylosis/discospondylosis	16 (5.5)	*Spondylosis*	*15 (0; 15)*
			*Discospondylitis*	*1 (0; 1)*
	Hip dysplasia	16 (5.5)	*Hip dysplasia*	*16 (0; 16)*
	Cruciate disease	6 (2.0)	*Cruciate disease*	*6 (0; 6)*
	Developmental skeletal disorder	5 (1.7)	*Incomplete ossification of the humeral condyle (IOHC)*	*2 (0; 2)*
			*Avascular necrosis of the femoral head (Legg-Calve-Perthes disease)*	*1 (0; 1)*
			*Carpal valgus*	*1 (0; 1)*
			*Limb deformity, developmental - genu valgum*	*1 (0; 1)*
	Arthropathy - elbow	4 (1.4)	*Elbow dysplasia*	*4 (0; 4)*
	Musculoskeletal mass/swelling	5 (1.7)	*Mass/swelling - mandibular*	*4 (0; 4)*
			*Mass/swelling - limb, lower*	*1 (0; 1)*
	Myositis	2 (0.7)	*Masticatory myositis*	*1 (0; 1)*
			*Myositis*	*1 (0; 1)*
	Skeletal neoplasia	1 (0.3)	*Osteochondroma - vertebral column*	*1 (0; 1)*
	Osteochondritis dissecans	1 (0.3)	*Osteochondritis dissecans*	*1 (0; 1)*
	Traumatic injury - tail	1 (0.3)	*Tail injury*	*1 (0; 1)*
	Arthropathy - carpus	1 (0.3)	*Joint instability - carpal*	*1 (0; 1)*
	Metaphyseal osteopathy (Hypertrophic osteodystrophy)	1 (0.3)	*Metaphyseal osteopathy (Hypertrophic osteodystrophy)*	*1 (0; 1)*
**Anal sac disorders** (172; 9.8%)	Anal sac impaction/infection	192 (95.5)	*Anal sac infection*	*129 (5; 124)*
			*Anal sac impaction*	*63 (4; 59)*
	Mass lesion - anal sac	7 (3.5)	*Mass lesion - anal gland/sac*	*7 (0; 7)*
	Anal sac disorder (unspecified)	2 (1.0)	*Anal sac disorder (unspecified)*	*2 (0; 2)*
**Respiratory disorders** (94; 5.4%)	Upper respiratory system disorder	65 (63.7)	*Infectious canine tracheobronchitis (Kennel Cough)*	*29 (1; 28)*
			*Brachycephalic airway obstruction syndrome (BAOS)*	*16 (0; 16)*
			*Respiratory tract infection, upper (URTI)*	*10 (1; 9)*
			*Tracheobronchitis finding*	*7 (0; 7)*
			*Tracheal collapse*	*2 (0; 2)*
			*Foreign body - nasal (nose)*	*1 (0; 1)*
	Lower respiratory system disorder	31 (30.4)	*Pulmonary oedema*	*28 (0; 28)*
			*Mass lesion - pulmonary (lung)*	*1 (0; 1)*
			*Neoplasm - pulmonary (lung)*	*1 (0; 1)*
			*Respiratory tract infection, lower (LRTI)*	*1 (0; 1)*
	Respiratory system disease (general)	6 (5.9)	*Respiratory tract infection (unspecified)*	*3 (0; 3)*
			*Chronic airway disease*	*1 (0; 1)*
			*Smoke inhalation*	*1 (0; 1)*
			*Parasite infestation-Aelurostrongylosis*	*1 (0; 1)*
**Herniation** (82; 4.7%)	Hernia	84 (100.0)	*Hernia - umbilical*	*72 (8; 64)*
			*Hernia - abdominal*	*5 (0; 5)*
			*Hernia - inguinal*	*4 (0; 4)*
			*Hernia (unspecified)*	*3 (2; 1)*

Magnetic Resonance Imaging (MRI) diagnoses of syringomyelia (SM), canine Chiari-malformation (CM) and both concurrently were recorded in 19 (1.1%; 95% CI 0.7 -1.7%), 4 (0.2%; 95% CI 0.07 - 0.6%) and 10 (0.6%; 95% CI 0.3 - 1.1%) study dogs, respectively. Thus, diagnosis of SM, CM or both was recorded in 33 (1.9%; 95% CI 1.3 – 2.7%) of study dogs overall. Syringomyelia ranked 28th among specific diagnoses most frequently recorded.

Meaningful statistical comparison of diagnostic frequencies between KC-registration status groups was not possible due to the relatively low numbers of KC registered dogs identified.

### Discussion

This study describes the most common diagnoses and disorder types recorded in nearly 2000 dogs identified as CKCSs in EPRs held by 151 UK primary-care veterinary clinics. The five specific disorders diagnosed in the greatest proportion of CKCSs during the study period were heart murmurs, diarrhoea of unspecified cause, dental disease, otitis externa and anal sac infection. The disorder categories affecting the greatest proportion of study dogs were cardiac disorders (largely due to the number of dogs with a recorded heart murmur), dermatological, ocular, gastrointestinal and dental/periodontal disorders.

Previous work has primarily focused on multi-breed canine populations and though not directly comparable to the current CKCS-specific findings, these earlier studies reported a number of similarities with respect to disease predispositions. A 2013 study reported that the most prevalent specific diagnoses across a random, multi-breed sample of dogs attending VetCompass-participating practices were otitis externa (10.2% of study dogs), periodontal disease (9.3%) and anal sac impaction (7.1%), with enteropathic disorders as the predominant disorder category [11]. Similarly, two other studies of primary-care canine caseloads of veterinary clinics in the US and UK also highlighted dental disease, otitis externa, anal sac impaction, diarrhoea and vomiting among the most common diagnoses in dogs [13,30]. Numerical differences in reported prevalence estimates between studies could reflect geographical variations or genuine changes in disease prevalence over time and could also be explained by fundamental differences in study design (e.g. calculating prevalence per consultation rather than per dog). However, alongside the present study, these findings indicate that certain health problems are common across a spectrum of canine breeds and types, including the CKCS. Prioritising clinical research to generate evidence-based recommendations pertinent to these conditions would therefore be of benefit to CKCSs but also to the wider canine population.

In the present study, cardiac disorders ranked highest in frequency at both the specific disorder and broad category levels, with murmurs recorded in 31%, unspecified heart disease in 7.3% and MVD in 5.0% of dogs with clinical entries during the study period. Heart disease was less frequently documented in similar, multi-breed studies with other breeds more strongly represented in the study sample [11,13,30]; for example, the level of cardiac disease in CKCSs appears much higher than the 5.6% prevalence reported by O’Neill et al. across all dog breeds [11]. This relative over-representation supports the importance of cardiac disease, and MVD in particular, as a prominent health issue in CKCSs [21,31-35] suggesting predispositions of the breed to cardiac conditions, including MVD [36].

Mechanisms of MVD inheritance continue to be investigated [22,36] but, given the importance of murmur detection in pre-breeding checks, further analysis of VetCompass data evaluating clinically relevant, murmur-related parameters and interactions (e.g. between murmur presence or grade and age) could inform protocols for diagnosis and routine monitoring of animals at the clinic level [37].

Far fewer KC registered dogs had reported murmurs compared to dogs of unknown registration status. It is possible that this finding reflects a genuinely lower prevalence of murmurs (and by implication existing or developing heart disease) in KC-registered CKCSs. Bias could have also been introduced (in either direction) by the comparative willingness of breeders to screen for heart murmurs in animals intended to produce puppies for KC registration, but it was not possible to explore this finding using the study data available.

Besides the dermatological component of congenital keratoconjunctivitis sicca and ichthyosiform dermatosis (CKCSID; commonly known as Dry Eye Curly Coat syndrome) [23], there is little evidence for inherited dermatological disorders of particular concern within the CKCS breed. In the present study, the most frequently reported specific skin disorders are similar to those in existing reports of multiple-breed canine veterinary caseloads [11,13,30,38] and include ectoparasites, unidentified cutaneous masses and skin infections. It is possible that availability of greater diagnostic detail in certain disorders (e.g., identification of unspecified cutaneous mass lesions or the underlying causes in pyoderma cases) would have highlighted disorders with established inherited components. However, the most frequent skin disorders noted in study dogs were generally not those linked to specific genetic defects in CKCSs, other than where typical conformational features can predispose to these conditions (for example, brachycephalic conformation pre-disposing to facial skin fold pyoderma).

Ocular disorders, and particularly corneal diseases, were frequently recorded in study dogs. KCS was particularly frequent, with a proportion of the unspecified corneal disorders (and chronic keratitis cases) possibly also due to undiagnosed KCS. Studies suggest an autosomal recessive mode of inheritance for CKCSID in the CKCS [23]. In addition, the typical CKCS skull morphology (with large eyes and shallow eye sockets) may also predispose the breed to corneal damage, exposure keratitis, conjunctival injury and subsequent irritation. Cataracts were recorded relatively frequently in study CKCSs and an inherited basis has been suggested for certain early onset cataracts in the breed [39,40]. However, the current study could not differentiate between inherited, early forms and age-related degenerative cataracts. DNA screening tests (e.g. CKCSID) and British Veterinary Association (BVA)/KC health schemes (e.g., multifocal retinal dysplasia and hereditary cataract) offer opportunities to reduce population levels of certain inherited conditions through selective breeding. However, the current study indicates that eye disorders remain an important challenge for those concerned with improving the health and welfare of CKCSs.

Gastrointestinal disorders also affected a notable number of study dogs, with diarrhoea of unspecified cause and other non-specific digestive disorders (e.g., gastroenteritis) frequently recorded. It is likely that a large proportion of the events recorded as non-specific gastroenteropathies were related to non-genetic causes such as dietary indiscretion, infection, parasitic disease or foreign body ingestion. However, existing studies report a high prevalence of chronic pancreatitis and exocrine pancreatic insufficiency (EPI) in CKCSs suggesting a heritable component of these conditions in the breed [41-43]. Batchelor notes that (in contrast to German Shepherd dogs) EPI is frequently not considered or tested for in CKCSs with digestive problems and may be under-recognised [42]. A proportion of the general diarrhoea and gastroenteropathies reported in the current study could thus be attributable to undiagnosed, breed-related chronic pancreatitis and/or EPI. Further investigation, perhaps using prospective study designs, is required to elucidate the relevance of inherited gastrointestinal conditions in this breed.

MRI-confirmed diagnoses of either syringomyelia, CM or both were recorded in approximately 2% of study dogs, with syringomyelia ranked 28th among the most frequently recorded specific diagnoses. Including only those diagnoses of either condition made with reference to MRI results (i.e. excluding those suggested by clinical signs alone) could have underestimated the true level in the population, as not all clinically suspicious cases underwent MRI scanning. The sometimes vague nature of clinical signs may result in failure to offer scanning in some cases, while reluctance (or financial limitations) of owners to pursue MRI diagnosis may be the limiting factor in others. Bias linked to comparative willingness to request high level diagnostic investigation may be of particular relevance in breeding animals, if breeders are more (or less) likely to volunteer breeding animals for MRI through screening programs [44]. The link between signs of SM/CM visible on MRI and appearance of clinical signs can also be inconsistent, as not all dogs with a visible syrinx on MRI display clinical signs at the time of imaging [45].

In the current study, data on sex, birth date and coat colour data were complete for over 99% of the study dogs, largely due to default settings within PMS software but perhaps also because this information is straightforward to ascertain or estimate when registering dogs with a clinic. However, many disorder presentations were clinically managed without a recorded precise diagnosis. In primary-care practice clinical, owner and resource-related factors (e.g. response to initial trial therapy, financial or time constraints, reluctance to present animals for re-examinations or continued treatment, limited-laboratory testing, post-mortem investigation and referral) can all limit confirmation of a definitive diagnosis. Recent, observational research in UK primary-care practice reported that, at a consultation level, a definitive diagnosis was recorded in only 21% of health problems encountered in companion animals [46], and a US cross-sectional study using EPR data recorded definitive diagnoses in only a third of small animal consultations in a primary-care setting [13].

#### Study limitations

The presence of dogs weighing up to 27kgs decreased confidence in some recorded breed categorisations, and confirmation of KC-registration via available microchip data was possible in only 4% of all dogs identified as CKCSs in the VetCompass database. The limited number of dogs for which KC registration could be confirmed also prevented meaningful statistical comparison between registered and unregistered subgroups regarding frequency of recorded disorders. A 2013 VetCompass study including all canine breeds reported a similarly low linkage rate, with 4.3% of 69,213 microchipped dogs confirmed as KC-registered despite identification as KC-recognised breeds [11]. Both studies encountered problems with availability and matching of dog identification data held in different databases. Confirmation of KC-registration for individual dogs required linkage using either microchip or KC registration codes between the VetCompass and KC registration databases. Barriers to linkage included non-universal microchipping of dogs in the general population, low recording proportions for KC registration details in EPRs and limited holding of microchip data in the KC registration database. Linkage success is expected to improve over the coming years as legislation requiring universal dog microchipping is enacted in various UK regions and will be further enhanced by improved veterinary and KC data recording processes.

Although the facility to record diagnostic terms using VeNom standardised diagnostic codes was available to the participating practices, the application of this coding option to report final diagnoses was not universal, thereby necessitating time-intensive manual searching of free-text clinical notes to extract diagnosis data. Wider (ideally universal) uptake and application of a standardised clinical nomenclature system, such as the VeNom codes [47], to routinely record clinical data in a consistent format would enhance practice-based research. Clinical coding would improve the efficiency of case-finding and other relevant data identification, reduce problems associated with misspellings, typing errors, ambiguous acronyms and abbreviations and perhaps reduce usage of outdated clinical terms, whilst retaining the ability to capture usage of valid synonyms for certain conditions.

At the time of this study, most VetCompass participating practices were part of a single practice group (Medivet Veterinary Group), located from north east to southern England, so it is possible that the findings of this study are less representative of situations elsewhere in the UK. While the study sample was randomly selected from all identified CKCSs, the authors did not attempt to stratify the sample to reflect overall geographical clinic distribution, potentially introducing an unquantifiable degree of geographical bias to the reported findings.

This study helps address knowledge gaps regarding disease prevalence in the UK population of a specific dog breed [6-8]. While many of the frequently recorded disorders do not have a specific, known inherited basis, genetic elements may contribute to their expression in CKCSs. Identifying the common disorders affecting CKCSs is an important step towards rational prioritisation of health issues within this breed [5,10]. Going forward, practical applications of this work could include consideration of breed-specific disorder prevalence estimates generated alongside associated welfare implications for affected dogs, ideally using a standardised system such as the Breed Disorder Welfare Impact Score [10]. This would allow comparison of the relative welfare impact of individual disorders on this population [4], placing commonly diagnosed conditions in the context of comparative welfare impact and highlighting conditions for prioritisation when allocating available resources for canine health research.

## Conclusions

These UK, breed-focused findings suggest that, while many of the disorders commonly affecting CKCSs are largely similar to those affecting the general dog population presented for primary-care veterinary services in the UK, cardiac disease (and MVD in particular) continues to be of particular concern in this breed. The study findings augment the evidence base available to clinicians managing CKCS patients, aid clinical decision-making and can inform health advice for owners (or prospective owners) of these dogs.

In future studies, confirmation of pedigree status in larger numbers of study dogs could improve the validity of breed-specific disease prevalence findings generated using primary-care practice EPR data. Further standardisation of veterinary terminology and disorder categorisation systems would facilitate more meaningful comparison, and potentially pooling, of findings from similar but independently conducted studies. Nonetheless, the current work contributes valuable evidence to inform prioritisation of breed-related disease issues in the CKCS, helping to direct future research and further focus efforts surrounding pre-breeding screening programmes for known inherited conditions.

## Methods

Ethics approval was granted by the Royal Veterinary College (RVC) Ethics and Welfare Committee (reference number 2010 1076B).

Study dogs were identified from a large database of EPR data (including clinical and demographic information) recorded by primary-care practice veterinary surgeons in English clinics participating in the VetCompass Animal Surveillance project [19]. VetCompass is an ongoing initiative enabling veterinary clinics to share routinely recorded, de-identified animal health data (including patient demography, clinical notes and prescribing data associated with healthcare episodes) for epidemiological research. Veterinary surgeons in participating clinics recorded free-text clinical information as normal and, in addition, were able to select clinical terms from a system of standardised veterinary nomenclature (the VeNom codes [47]) embedded within their practice management software (PMS) to describe diagnoses at clinical encounters. Clinical queries integrated within the PMS enabled regular extraction of clinical care data from the computerised records of participant clinics. These data were automatically uploaded into the main VetCompass structured query language (SQL) database, held on a secure RVC server. Data collected via this system included animal identification (ID) number, patient demographic information (e.g., species, breed, date of birth, sex, neuter status, microchip number and bodyweight), clinical information (free-text clinical notes and any diagnostic codes assigned) as well as treatment and prescribing details, all with relevant dates.

The study sampling frame included all dogs with breed recorded as CKCSs within the VetCompass database at the time of the study query (i.e., dogs registered with participating clinics at any time up until the 9th July 2013). For dogs with a recorded microchip number, Petlog microchip and KC-registration databases were cross-queried; KC-registration status was classified as either ‘confirmed’ or ‘unknown’ based on available information. Sample size calculations estimated that, from a study population of 3624 dogs (i.e., all dogs from the VetCompass database with CKCS as recorded breed), a sample of 1843 animals (51% of the available study population) would be required to represent a disorder with 2.5% expected frequency and a precision of 0.5% (95% confidence level, 80% power) [48].

The study sample was formed by randomly selecting 52% of the dogs in each KC-registration subgroup from the overall sampling frame, using the random number generation function in Microsoft Excel (Microsoft Office Excel 2007, Microsoft Corp.). The study group thus contained equal proportions of the overall identified CKCSs with ‘confirmed’ and ‘unknown’ KC-registration status combined to be representative of the overall KC registration status ratio. Restriction of the study sample to a subset of the overall identified CKCSs was necessary because of the time-intensive nature of reviewing full clinical histories in detail.

Clinical notes and VeNom diagnosis terms entered during the study period were reviewed in detail for all study dogs. The most definitive (or clinically specific) diagnostic term used for each disorder recorded in individual animals was manually coded by assigning the most appropriate term from the VeNom list. Clinical events considered entirely prophylactic or elective (e.g., vaccination or neutering, respectively) were not included. Recurring diagnoses of ongoing conditions were included only once in individual dogs, using the final diagnosis term recorded over time if multiple terms were used during the study period (following diagnosis revision, confirmation by diagnostic testing, or clinical progression). This approach aimed to avoid multiple counting of transient but recurrent disorders in ongoing cases, and assumed that diagnostic certainty generally increased over time [11].

No distinction was made between diagnoses recorded in association with ongoing disorders (pre-existing conditions) and those newly diagnosed during the study period (incident conditions). Disorders specified within the clinical notes only to the level of presenting sign terms (e.g., vomiting, diarrhoea, or cardiac murmurs of specified or unspecified grade, without recording of a formal diagnostic term) were included as such, to represent the best available indicator for the clinical disorder documented. Dental disorders were included only when severe enough to result in a veterinary recommendation for medical or surgical intervention.

Data on patient demography (bodyweight, age, colour and sex) were extracted from the VetCompass database for all study dogs. The maximum recorded bodyweight value was extracted for each dog aged 9 months or older, to best reflect mature bodyweight. Age, in years, at earliest and latest recorded EPR entry for individual dogs was calculated, with the minimum, maximum and median ages at these points presented for the study group as a whole, and for subgroups according to KC-registration status. Coat colour was categorised as Blenheim, tri-colour, ruby, black and white, black and tan, other solid colour, other mixed colour or unspecified. Time contributed to the study was defined for individual dogs as the period from the first available EPR entry to the last in the study period or date of recorded death.

VeNom diagnostic terms used to code recorded disorders (referred to in this paper as ‘specific disorder’) were extracted for each dog and for each diagnostic term the number of study dogs affected during the study period was described. Specific disorders were also mapped to two systems of terms, to allow presentation of results at differing levels of precision and potentially indicate disorder types or body systems of particular importance in study dogs. Thus, specific disorder level terms were the original coded terms as extracted from the clinical notes, reflecting the maximum diagnostic precision recorded for each disorder in each individual animal. These descriptors were mapped into broad ‘disorder categories’ (largely based on primary body system affected) and into ‘disorder groups’ within these categories; the latter allowed expression of some originally extracted terms at a more general level of diagnostic precision while bringing together disorders of similar types within a body system. For example, the specific recorded diagnostic term *‘Parasite infestation – fleas’* would be classified as *‘Ectoparasite infestation’* at the disorder group level, within the dermatological disorders category. The study unit of interest was disorders diagnosed, therefore in dogs with recorded diagnosis of multiple different disorders, all disorders (rather than a single, arbitrarily selected ‘primary complaint’ per dog) were classified according to the systems described.

Data for the study were exported from the VetCompass SQL database to Microsoft Excel (Microsoft Office Excel 2007, Microsoft Corp.) for reformatting and cleaning. Spreadsheets produced were then exported to Stata Version 11.2 (Stata Corp.) for descriptive statistical analysis. The distribution (with median values for continuous variables) of sex, coat colour, age at first and last EPR entry, and time contributed to the study was presented for all study dogs, and by subgroup according to UK KC-registration status. Dogs with available demographic data but no clinical EPR entries during the study period were identified and counted.

For each disorder category, the number of dogs affected by at least one included condition, and the number of disorder recordings within each smaller disorder group were counted. Prevalence estimates for specific disorders in the UK CKCS population were calculated, based on the proportion of study dogs affected during the study period. A GraphPad Software online QuickCalc calculator (GraphPad Software, http://www.graphpad.com/quickcalcs/ConfInterval1.cfm (accessed May 2014)) was used to calculate 95% confidence intervals (CIs) for prevalence estimates, via a modified Wald method.

## Abbreviations

BOAS: Brachycephalic obstructive airway syndrome
BDWIS: Breed-disorder welfare impact score
BVA: British Veterinary Association
CI: Confidence interval
CKCSID: Congenital keratoconjunctivitis sicca and ichthyosiform dermatosis syndrome
CKCS: Cavalier King Charles Spaniels
CM: Canine Chiari-malformation
CMSM: Canine Chiari-malformation/syringomyelia
DJD: Degenerative joint disease
EPR: Electronic patient record
HGE: Haemorrhagic gastroenteritis
ID: Identification
KC: The Kennel Club
KCS: Keratoconjunctivitis sicca
KCCT: Kennel Club Charitable Trust
MRI: Magnetic resonance imaging
MVD: Mitral valve disorder/disease/degeneration
PMS: Practice management software
RA: Rheumatoid arthritis
RVC: Royal Veterinary College
SQL: Structured query language
SM: Syringomyelia
UK: United Kingdom
URTI: Upper respiratory tract infection
UTI: Urinary tract infection
VeNom: Veterinary nomenclature
